# Epidemiological and Clinical Characteristics, Antifungal Susceptibility, and MLST-Based Genetic Analysis of *Cryptococcus* Isolates in Southern Taiwan in 2013–2020

**DOI:** 10.3390/jof8030287

**Published:** 2022-03-11

**Authors:** Yi-Chun Chen, Shu-Fang Kuo, Shang-Yi Lin, Yin-Shiou Lin, Chen-Hsiang Lee

**Affiliations:** 1 Division of Infectious Diseases, Department of Internal Medicine, Kaohsiung Chang Gung Memorial Hospital, Kaohsiung 83301, Taiwan; sonice83@yahoo.com.tw (Y.-C.C.); shiou0428@cgmh.org.tw (Y.-S.L.); 2 Department of Laboratory Medicine, Kaohsiung Chang Gung Memorial Hospital, Kaohsiung 83301, Taiwan; ivykuo@cgmh.org.tw; 3 Department of Medical Biotechnology and Laboratory Sciences, College of Medicine, Chang Gung University, Taoyuan 33302, Taiwan; 4 Division of Infectious Diseases, Department of Internal Medicine, Kaohsiung Medical University Hospital, Kaohsiung 80756, Taiwan; amoe616@gmail.com; 5 Department of Laboratory Medicine, Kaohsiung Medical University Hospital, Kaohsiung 80756, Taiwan; 6 College of Medicine, Kaohsiung Medical University, Kaohsiung 80708, Taiwan; 7 School of Medicine, College of Medicine, Chang Gung University, Taoyuan 33302, Taiwan

**Keywords:** cryptococcosis, cryptococcemia, molecular typing, azole, flucytosine, amphotericin B, mortality

## Abstract

Cryptococcal meningoencephalitis (CM) is a treatable condition, but it leads to excessive morbidity and mortality. We collected 115 non-duplicated *Cryptococcus* clinical isolates during 2013–2020 in southern Taiwan to perform antifungal susceptibility testing. Multi-locus sequence typing was performed on 96 strains from patients with CM (*n* = 47) or cryptococcemia (*n* = 49). In addition, the epidemiological and clinical characteristics of patients with CM during 2013–2020 (*n* = 47) were compared with those during 2000–2010 (*n* = 46). During 2013–2020, only one *C. neoformans* isolate (0.9%) had a fluconazole minimum inhibitory concentration of >8 μg/mL. Amphotericin B (AMB), flucytosine (5FC), and voriconazole were highly active against all *C. neoformans*/*C. gattii* isolates. The most common sequence type was ST5. Among these 47 patients with CM, cerebrospinal fluid cryptococcal antigen (CSF CrAg) titer >1024 was a significant predictor of death (odds ratio, 48.33; 95% CI, 5.17–452.06). A standard induction therapy regimen with AMB and 5FC was used for all patients during 2013–2020, but only for 2.2% of patients in 2000–2010. The in-hospital CM mortality rate declined from 39.1% during 2000–2010 to 25.5% during 2013–2020, despite there being significantly younger patients with less CSF CrAg >1024 during 2000–2010. The study provides insight into the genetic epidemiology and antifungal susceptibility of *Cryptococcus* strains in southern Taiwan. The recommended antifungal drugs, AMB, 5FC, and FCZ, remained active against most of the *Cryptococcus* strains. Early diagnosis of patients with CM and adherence to the clinical practice guidelines cannot be overemphasized to improve the outcomes of patients with CM.

## 1. Introduction

Cryptococcosis, a potentially fatal mycosis worldwide, is caused by members of the *Cryptococcus neoformans* and *C. gattii* species complexes [1,2]. Although *C. neoformans* and *C. gattii* share many features of a highly evolved, environment-savvy yeast, there are important species- and strain-specific differences related to geographical distribution, environmental niches, host preference, and clinical manifestations [1]. Cryptococcosis encompasses a spectrum that ranges from latent infection through subclinical disseminated disease to fulminant meningoencephalitis. Even in middle- and high-income countries, cryptococcosis-associated mortality remains high [3,4]. Early diagnosis, efficient clinical support, and proper antifungal therapy are essential factors to reduce the mortality and adverse impacts of cryptococcosis [5].

Cryptococcal meningoencephalitis (CM) is characterized by higher mortality than non-central nervous system (CNS) cryptococcosis and substantial long-term neurological sequelae post-treatment [6]. CM often involves patients with advanced human immunodeficiency virus (HIV) disease, malignancy, and immunosuppressive conditions, such as recipients of solid-organ transplants. It also occurs in immunocompetent hosts [1]. The use of potent antiretroviral therapy has significantly reduced the incidence of CM in people living with HIV in developed countries [7]. In clinical practice, the antifungal treatment of CM involves sequential therapeutic phases, including induction, consolidation, and maintenance phases. A course of amphotericin B (AMB) with flucytosine (5FC) followed by fluconazole (FCZ) as consolidation and maintenance is considered as the benchmark in antifungal therapy for CM [8]. Additionally, liposomal amphotericin B (LAmB) is a preferred alternative to conventional AMB, with similar outcomes and less nephrotoxicity [9], and is recommended specifically for primary induction in patients at risk of renal dysfunction.

Numerous reports have been published on the *Cryptococcus* spp. isolates with high resistance to FCZ [10,11]. FCZ-non-susceptible *Cryptococcus* spp. isolates have been reported in cases of treatment failure mainly associated with acquired immunodeficiency syndrome (AIDS) [10,12]. Several factors have been found to affect the susceptibility of *Cryptococcus* spp. isolates to antifungal drugs, which include the prolonged use of FCZ as a suppressive monotherapy, low bioavailability of FCZ in the infected tissue, fungistatic action of azoles, increased tolerance to FCZ due to replicative aging, low glucose growth condition and limited nutrients, and intrinsic resistance, such as the hetero-resistance of *C. neoformans* and *C. gattii* to azoles [11,13,14,15]. The environmental triazole (tebuconazole) used as an agrochemical pesticide may induce cross-resistance to clinically available azoles in *C. neoformans* and *C. gattii* [16]. Moreover, it is noteworthy that a high frequency of *C. neoformans* isolates with non-wild-type (non-WT) susceptibility to AMB has been reported [17,18]. However, routine antifungal susceptibility testing (AST) is not performed in most microbiological laboratories. With geographical variations, the epidemiological surveillance of the antifungal resistance of cryptococcal strains is crucial for clinical practice.

In the last few decades, several widely used molecular methods have been used worldwide to determine the genotypes of clinical, environmental, and veterinary isolates of the members of the *C. neoformans* and *C. gattii* species complexes to investigate their geographical distribution, molecular epidemiology, and population genetics [19,20,21,22,23]. Understanding the epidemiological and microbiological characteristics of local *Cryptococcus* strains and clinical features of CM is essential for the development of efficient diagnosis and treatment strategies. The purpose of the present study was to determine the antifungal susceptibility of clinical strains of *C. neoformans* and *C. gattii* collected in southern Taiwan to AMB, 5FC, FCZ, posaconazole (PCZ), and voriconazole (VCZ) using standard methods (Clinical and Laboratory Standards Institute, CLSI M27-A3 broth microdilution) [24]. Multi-locus sequence typing (MLST) was carried out on *C. neoformans* and *C. gattii* species complex isolates from patients with cryptococcosis. The clinical and demographic features and outcomes of patients with CM during 2013–2020 were reviewed and compared with those in our previous study (2000–2010) [25].

## 2. Materials and Methods

### 2.1. Cryptococcus Strains

During 2013–2020, 115 isolates of *Cryptococcus* strains (109 *C. neoformans* and 6 *C. gattii*) were identified from 115 patients admitted to Kaohsiung Chang Gung Memorial Hospital (KCGMH) (92 isolates) and Kaohsiung Medical University Hospital (KMUH) (23 isolates), Southern Taiwan. These isolates were cultured from blood (49), cerebrospinal fluid (CSF) (47), respiratory specimens (16), and urine specimens (3). The isolated strains were preserved at −70 °C. The sample processing and identification of isolates were performed by matrix-assisted laser desorption/ionization time–of–flight mass spectrometry (Microflex LT, Bruker Daltonik GmbH, Bremen, Germany).

### 2.2. Antifungal Susceptibility Testing (AST)

The broth microdilution method was performed according to the recommendations of the CLSI in the M27-A3 protocol [24]. Antifungal agents were obtained as reagent-grade powders with high purity (Sigma, Schnelldorf, Germany). Three azoles, including FCZ, VCZ, and PCZ, AMB, and 5FC were diluted according to the CLSI standards. The medium used in the assays was Roswell Park Memorial Institute (RPMI) 1640, which had been buffered with 165 mM MOPS to pH 7.0 (Sigma-Aldrich^®^). Tests were performed in sterilized 96-well, flat-bottomed microplates with lids. These were inoculated with 5.0 × 10^2^–2.5 × 10^3^ cells/mL and incubated at 35 °C for 72 h without shaking. For the azoles and 5FC, the minimum inhibitory concentrations (MICs) were recorded as the lowest concentration that inhibited the fungal growth by 50% compared to the control. The MIC for AMB was the lowest concentration resulting in an optically clear zone. *C. parapsilosis* (ATCC 22019) and *C. krusei* (ATCC 6258) served as control strains in all test plates. AST was repeated for all strains at least twice at different times to check the strain stability and reproducibility of the MIC results.

### 2.3. Result Interpretation

Epidemiological cut-off values (ECVs) are the MIC/minimal effective concentration values that segregate the fungal populations into those with and without acquired and/or mutational resistance based on AST results. The ECV MIC values proposed for *C. neoformans* VNI are as follows: AMB 0.5 μg/mL, 5FC 8 μg/mL, FCZ 8 μg/mL, VCZ 0.25 μg/mL, and PCZ 0.25 μg/mL [26]. The ECVs of *C. gattii* VGI and VGII were determined according to the recommendations of Espinel-Ingroff et al. [26]. Strains with MIC values lower than or equal to the ECVs were classified as wild-type (WT), whereas those with higher values than the ECVs were non-WT.

### 2.4. Multi-Locus Sequence Typing (MLST) and Phylogenetic Analysis

MLST for seven genetic loci, including *CAP59*, *GPD1*, *LAC1*, *PLB1*, *SOD1*, *URA5*, and the IGS1 region, was performed using the International Society of Human and Animal Mycology consensus MLST scheme for *C. neoformans s.l.* and *C. gattii s.l.* The seven loci were amplified using the primers listed on the website of the Fungal MLST Database (http://mlst.mycologylab.org, 24 June 2021). Sequences for each locus were assigned an allele type number. Thereafter, on accessing the MLST database to combine the allele types, several sequence types (STs) were obtained. Molecular types (i.e., VNI to VNIV for the *C. neoformans* species complex and VGI to VGIV for the *C. gattii* species complex) were assigned according to their allelic numbers and STs. Phylogenetic analysis depicting the genetic relationships between isolates based on MLST locus alleles was conducted using the categorical analysis method, and minimum spanning tree analysis was performed using BIONUMERICS software (version 7.5, Applied Maths, Kortrijk, Belgium) based on the ST profiles of strains. To place the *C*. *neoformans* VNI population of Taiwan in the global context, MLST loci alleles from the clinical isolates were aligned with those of other *C*. *neoformans* VNI STs available in the Fungal MLST Database.

### 2.5. Geographical Distribution of STs

The residential addresses of the cases with CM or cryptococcemia and known STs were recorded according to districts, followed by mapping using SuperGIS Desktop software (Supergeo Technologies Inc., Taipei, Taiwan).

### 2.6. Retrospective Cohort Study

We conducted a retrospective cohort study to obtain information on the clinical characteristics and outcomes of patients with CM during 2013–2020 at KCGMH. The demographic characteristics, clinical findings, predisposing conditions, time of presentation to diagnosis, antifungal therapy, surgical intervention, and outcomes were recorded. Additionally, the characteristics and outcomes of patients with CM during 2000–2010 [25] and 2013–2020 were compared. The study was approved by the Institutional Review Board of Chang Gung Memorial Hospital (No. 201901403B0D001).

### 2.7. Statistical Analysis

Descriptive statistical methods were used to summarize the demographic characteristics, and outcome analysis was based on the inpatient mortality (death during hospitalization after CM diagnosis). *p*-values were calculated by Fisher’s exact test for categorical variables and Student’s t or Mann–Whitney U tests were used for continuous variables. Factors with a *p*-value of <0.1 in univariate analyses were entered into a multiple logistic regression model to identify independent predictors of in-patient mortality. All statistical tests were 2-tailed and significance was set at α = 0.05. Analyses were performed using SPSS 17.0 for Windows (SPSS Inc., Chicago, IL, USA).

## 3. Results

### 3.1. In Vitro Antifungal Susceptibility

The MIC ranges, MIC_50_, MIC_90_, and geometric mean (GM) of the five antifungal agents investigated against 109 *C. neoformans* and 6 *C. gattii* isolates are summarized in Table 1. The MIC for FCZ against *C. neoformans* ranged from 0.25 to 16 μg/mL (GM 2.55 μg/mL). Table 2 shows the distribution of the MIC values of five antifungal agents against *C. neoformans* and *C. gattii* strains. Only one isolate (0.9%) had an FCZ MIC >8 μg/mL. AMB, 5FC, and VCZ were highly active against all *C. neoformans* and *C. gattii* strains. Only one *C. neoformans* isolate (0.9%) had PCZ MIC > 0.25 μg/mL. Our results demonstrated that all six *C. gattii* strains were uniformly WT to AMB, FCZ, 5FC, and VCZ.

### 3.2. Molecular and Sequence Types

**Figure 1:**
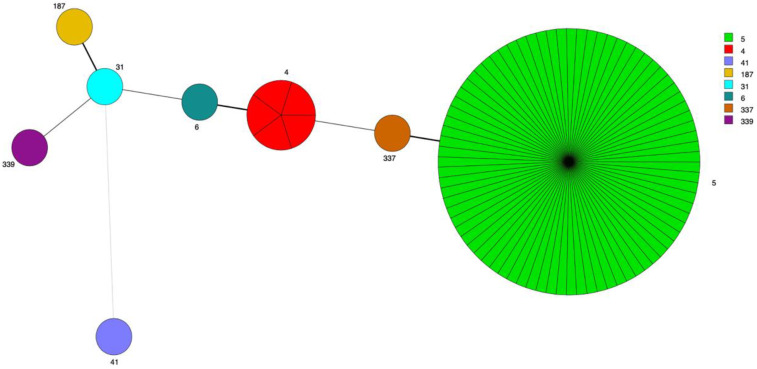
Population structure of clinical isolates of *C. neoformans* in Taiwan (Multilocus sequence typing). Minimum-spanning tree of the 8 detected sequence types and their relative distribution of 90 clinical isolates. The circle sizes are proportional to the numbers of isolates. The numbers near the circles represent the sequence types.

MLST was performed on 96 strains (90 *C. neoformans* and 6 *C. gattii*). The strains were classified into four groups based on the results as follows: 89 strains (92.7%) of *C. neoformans* were identified as genotype VNI and one (1.0%) as genotype VNII; and four strains (4.2%) of *C. gattii* were identified as genotype VGII and two (2.1%) as genotype VGI. MLST analysis identified eight different STs of *C. neoformans* and three STs of *C. gattii*. A total of 79 strains (82.3%) belonged to ST5, five (5.2%) to ST4, one (1.0%) to ST337, one to ST6, one to ST31, one to ST187, one to ST339, and one to ST41 (VNII; Figure 1). Of the six *C. gattii* strains, three (3.1%) belonged to ST7, two to ST106, and one to ST274. High diversity of *C. neoformans* STs (five different STs) was found in the strains isolated in the year 2020, and all eight STs were identified in the Kaohsiung–Pingtung region (Southern Taiwan) (Supplementary Figure S1).

### 3.3. Phylogenetic Analysis

For *C. neoformans* VNI, the dominant ST in Taiwan (ST5) was distributed worldwide, as it was isolated from China, the United States, Latin America, and European countries. The second common ST4 was reported from Asian and African countries. Of the other STs identified in this study, ST337 was also isolated from China, ST6 and ST31 were prevalent in Asian countries, ST339 was found in Vietnam, and ST187 was found in Uganda (Supplementary Figure S2).

### 3.4. Clinical Cohorts

During 2013–2020, 47 patients received a diagnosis of CM at KCGMH. All of them received standard induction therapy with AMB combined with 5FC. The in-hospital mortality was 25.5%. The duration of follow-up is shown in Supplementary Figure S3. The demographic characteristics, CSF cryptococcal antigen (CrAg) titers, and immunosuppressive conditions of survivors and non-survivors are summarized in Table 3. In univariate analysis, patients who survived CM were younger (mean age, survival vs. non-survival, 58 ± 18 versus 73 ± 12; *p* < 0.01) and less likely to have CSF CrAg titers > 1024 (17.1% vs. 83.3%; *p* < 0.01), and had a shorter duration from presentation to diagnosis (6.6 ± 7.1 days vs. 12.0 ± 11.5 days, *p* = 0.06). Patients with known immunocompromised status did not show a higher mortality rate compared to those without known immunocompromised status (*p* = 0.11). In the multivariable models, CSF CrAg titers > 1024 remained significantly associated with mortality (adjusted odds ratio, 48.33; 95% CI, 5.17–452.06).

Twenty-seven (57.4%) patients were treated with LAmB, including 8 (17.0%) who initiated LAmB and 19 (40.4%) who were switched from AMB due to adverse effects (median AMB treatment duration before switching to LAmB, 8 days [IQR, 4–14]). A total of 16 patients (34.0%) underwent surgical intervention to control increased intracranial pressure or hydrocephalus, including four external ventricular drainages (EVDs) alone, seven ventriculoperitoneal shunting (VP shunting), and five EVDs followed by VP shunting. Of the patients who survived CM, the mean duration of induction therapy was 27.4 ± 11.7, days and that of consolidation and maintenance therapies was 262.6 ± 249.0 days.

**Figure 2:**
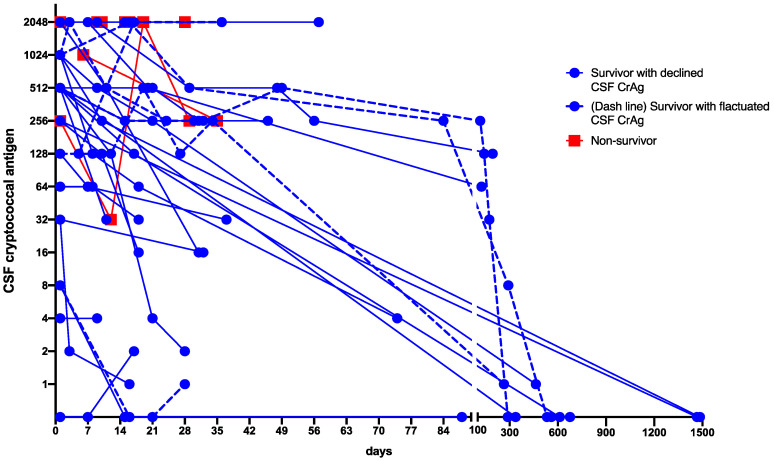
Evolution of CSF CrAg titers in 34 patients who had more than 1 determination of CrAg titers according to the day of diagnosis. CSF CrAg titer > 1024 is presented as 2048. CSF, cerebrospinal fluid; CrAg, cryptococcal antigen.

CSF CrAg titers were determined more than once in 34 of 47 (72.0%) patients with CM (Figure 2). Although CSF CrAg titers declined with time, six patients had positive CSF antigen titers that lasted for 3 months or longer after diagnosis. We further observed a higher mortality rate in patients with elevated or unchanged CrAg titers during treatment within 6 weeks after diagnosis than those with declining CrAg titers (5/11 (45.5%) and 1/23 (3.7%), respectively; *p <* 0.01).

During 2000–2010 [25], 46 patients were diagnosed with CM at the same hospital. Table 4 presents comparisons of the demographic and clinical characteristics of patients with CM during the two study periods (2000–2010 vs. 2013–2020). The in-hospital mortality was higher during 2000–2010 (39.1%) than 2013–2000 (25.5%) (*p =* 0.16). Compared to those in 2013–2020, the patients with CM in 2000–2010 were significantly younger (mean age, 51 ± 19 vs. 62 ± 18; *p* < 0.01) and less likely to have CSF CrAg titer >1024 (21.7% vs. 34.0%, *p =* 0.16). During 2000–2010, only 2.2% of the patients received standard induction therapy, 50.0% received AMB with FCZ, 37.0% received AMB alone, and 10.9% received FCZ alone.

## 4. Discussion

In the present study, we found that the clinical *C*. *neoformans* and *C. gattii* isolates obtained during 2013–2020 were susceptible to AMB, 5FC, and FCZ. The emergence of *Cryptococcus* strains with resistance or elevated MICs of antifungals above the ECVs is of great concern. According to a report that summarized 21 studies with 11,049 clinical *Cryptococcus* isolates, 11.4% of the isolates had FCZ MICs > 8 μg/mL; however, a substantial geographical difference was observed (non-WT range, 0–76.3%) [11]. A systemic review of 29 studies from 1988 to 2017 reported baseline FCZ resistance in 12% of *Cryptococcus* isolates, approximately 10% for incident isolates, and 24% for relapses, though definitions of resistance were different among the studies. Resistance to FCZ appears to be an important issue in *Cryptococcus* isolates from patients with relapses [10]. According to studies from China, provinces with a higher prevalence (61.9%) of HIV-related cryptococcosis reported higher non-WT rates to AMB and FCZ in *Cryptococcus* strains compared to those with lower prevalence (4.5%) [18,27]. In the report of a multicenter retrospective study of patients with proven cryptococcosis in Taiwan during 1997–2010 [28], only 0.5% (1/216) of *Cryptococcus* clinical isolates belonged to FCZ non-WT strains. In the study conducted in a region with lower prevalence (9/115, 7.8%) of HIV-related cryptococcosis, FCZ remained active against *Cryptococcus* isolates.

Cryptococcosis in southern Taiwan is mainly caused by *C. neoformans,* and more than 80% of the strains belong to ST5, the predominant ST in China (>90% of the strains belong to ST5) [29]. In Vietnam, strains belonging to ST5 caused cryptococcal meningitis in 35% (34/98) and 82% (31/38) of patients with and without HIV infection, respectively [30]. In contrast, ST93 was the most frequently identified ST in clinical isolates in Brazil [31]. Additionally, the highest diversity of *C. neoformans* STs was found in 2020 (five different STs). According to a study, 98.1% (206/210) of the clinical isolates from Taiwan showed the VNI genotype of *C. neoformans* and only 1.9% (4/210) were VNII [28]. Another variant ST41 (VNII genotype, also identified in Japan [32], South Africa, and the United States) was identified from an HIV-positive patient. The distribution of genotypes across various geographical locations may be related to weather and pigeons, animals, plants, and human activities [18]. In this study, around 10% of CM cases were caused by *C. gattii* during 2000–2010 and 2013–2020 at KCGMH. Lin et al. reported that, although no environmental *C. gattii* isolates were found in Pingtung, ST630 was commonly identified in Kaohsiung [19]. However, no ST630 clinical isolates were found in Taiwan, according to Lin et al. and the current study. Only one patient from Pingtung City was infected with *C. gattii* ST106.

The levels of CrAg titer provide good prognostic information. Initial high titers (≥1:1024) demonstrate a high burden of yeasts in the host, poor host immunity, and a high probability of therapeutic failure [33]. We noticed that an initial CSF CrAg titer > 1024 remained an independent risk factor of mortality when all patients received standard induction therapy. Despite being an excellent diagnostic test and a predictor of prognosis, the use of the CrAg test is presumed inaccurate in decision-making during antifungal treatment. Therefore, the use of serial polysaccharide antigen titers is not recommended to develop treatment guidelines [34]. However, more studies are required to understand the dynamics of clearance and prognostic utility in different disease states [6]. We observed that constant CrAg titers during treatment increased in-hospital mortality significantly.

We found that the mortality rate had declined from 2000–2010 to 2013–2020, despite the fact that patients with CM treated during 2013–2020 were older and had higher CSF CrAg titers (>1024) than those during 2000–2010. However, the difference in the mortality rates of patients with CM during the two periods was not statistically significant, as the case numbers were small for the two cohorts and 50% (6/12) of the mortality occurred within 14 days of hospitalization in the 2013–2020 cohort (Supplementary Figure S3). While cryptococcosis can be present in both immunocompromised and immunocompetent subjects, the immunocompromised status of patients with CM was not a risk factor of death in the present study. Late presentation and delayed diagnosis of patients with CM could be an important factor related to subsequent poor neurological outcomes [5]. We also observed that the duration between presentation to diagnosis was longer in patients with CM who died than in those who survived. All patients with CM during 2013–2020 received standard induction therapy according to the Infectious Diseases Society of America (IDSA) documented guidelines on the management of cryptococcal disease [35]; however, only 2.2% of the patients with CM received this treatment during 2000–2010 (*p* < 0.001). The results suggested that the inclusion of 5FC in the induction therapy had an impact on the mortality of CM. AMB used to be the first-line treatment in non-organ-transplant patients without renal function impairment [36]. Nevertheless, it was observed that 40% of the patients with CM required switching to LAmB due to adverse effects and intolerance. In resource-available areas, the liposomal product has become the preferred polyene [1], though comparative studies with 5FC combined with lipid formulations of AMB as opposed to AMB remain scarce.

Despite various key findings, this study has several limitations. We collected and analyzed *Cryptococcus* isolates and patients with CM from southern Taiwan. Multicenter studies are necessary to understand the microbial and clinical epidemiological characteristics of the whole country. Moreover, we described only in-hospital mortality as a clinical outcome, and the long-term neurological sequelae could not be assessed due to the intrinsic limitations of the retrospective study. The outcomes after discharge were not known for some patients due to the unavailability of description in medical records or loss to follow-up. The case numbers of the two cohorts of patients with CM were small, which might preclude us from observing statistically significant differences in the overall mortality rates, though the mortality rate in the 2013–2020 cohort was numerically lower than that in the 2000–2010 cohort. While adherence to the clinical treatment guidelines [35] in initiating standard induction therapy for CM could have contributed to the lower mortality observed in this study, we were not able to examine the impact of clinical experience and evolution of management of CM on the outcomes of the later cohort. However, early mortality remained high in our included patients with CM, probably related to poor host condition and late presentation of the patients with CM during the two study periods.

## 5. Conclusions

This study provides insight into the genetic epidemiology and antifungal susceptibility of *Cryptococcus* strains in southern Taiwan. The recommended antifungal drugs, AMB, 5FC, and FCZ, remained active against most of the *Cryptococcus* strains. The most common sequence type was ST5. Early diagnosis of patients with CM and adherence to clinical practice guidelines cannot be overemphasized to improve the outcomes of patients with CM.

## Figures and Tables

**Table 1:** In vitro susceptibility of clinical isolates of *Cryptococcus neoformans* and *C. gattii* to five antifungal agents as determined by CLSI broth microdilution.

*Cryptococcus* Species, No. of Isolates, Antifungal Agent	MIC (μg/mL)		
	Broth Dilution		
	GM	MIC_50_/MIC_90_	Range
***C. neoformans***, N = 109			
Amphotericin B	0.09	0.06/0.25	0.03–0.5
Flucytosine	1.31	1/2	0.5–4
Fluconazole	2.55	2/4	0.25–16
Posaconazole	0.04	0.03/0.25	0.015–0.5
Voriconazole	0.05	0.06/0.12	0.015/0.25
***C. gattii***, N = 6			
Amphotericin B	0.10	0.06/0.25	0.06–0.25
Flucytosine	1.12	1/4	0.25–4
Fluconazole	1.78	1/8	0.5–8
Posaconazole	0.10	0.06/0.5	0.015–0.5
Voriconazole	0.07	0.12/0.25	0.015–0.25

Abbreviations: GM, geometric mean; MIC, minimal inhibitory concentration; N, number. MIC50 and MIC90, MICs at which 50% and 90% of the isolates were inhibited.

**Table 2:** Distribution of the MIC values using CLSI broth microdilution for clinical strains of *Cryptococcus neoformans* and *C. gattii*.

Species (No. of Isolates)	Antifungal Agent	N of Isolates with MIC (μg/mL) of the Tested Antifungal Agents										
		0.015	0.03	0.06	0.12	0.25	0.5	1	2	4	8	16
*C. neoformans* (N = 109)	Amphotericin B		13	44	36	14	2					
	Flucytosine						11	50	42	6		
	Fluconazole					1	4	9	44	45	5	1
	Posaconazole	33	26	23	11	15	1					
	Voriconazole	6	30	62	9	2						
*C. gattii* (N = 6)	Amphotericin B			3	2	1						
	Flucytosine					1		3	1	1		
	Fluconazole						2	1		2	1	
	Posaconazole	1	1	1	1		2					
	Voriconazole	2			3	1						

Abbreviations: MIC, minimal inhibitory concentration; N, number.

**Table 3:** Demographic and clinical characteristics of 47 patients with cryptococcal meningoencephalitis.

	All	Survivors	Non-Survivors	*p*-Value
Total No. (%)	47 (100)	35 (74.5)	12 (25.5)	
Age (mean ± SD), years	62 ± 18	58 ± 18	73 ± 12	<0.01
Female sex	14 (29.8)	9 (25.7)	5 (41.7)	0.47
Time to diagnosis from presentation (mean ± SD), days	8.0 ± 8.6	6.6 ± 7.1	12.0 ± 11.5	0.06
CSF CrAg titer > 1024	16 (34)	6 (17.1)	10 (83.3)	<0.01
Cryptococcemia	14 (29.8)	8 (22.9)	6 (50.0)	0.14
*C. gattii*	6 (12.8)	6 (17.1)	0	0.32
HIV infection	4 (8.5)	4 (11.4)	0	0.56
Hematologic disease/malignancy	7 (14.9)	5 (14.3)	2 (16.7)	>0.99
Liver cirrhosis	4 (8.5)	2 (5.7)	2 (16.7)	0.27
Solid-organ transplantation	2 (4.3)	2 (5.7)	0	>0.99
Solid-organ malignancy	8 (17.0)	5 (14.3)	3 (25.0)	0.40
Autoimmune disease	5 (10.6)	3 (8.6)	2 (16.7)	0.59
Known immunocompromised status	26 (55.3)	17 (48.6)	9 (75.0)	0.11

Abbreviations: CrAg, cryptococcal antigen; CSF, cerebrospinal fluid; HIV, human immunodeficiency virus; SD, standard deviation.

**Table 4:** Comparisons of demographic and clinical characteristics of two cohorts with cryptococcal meningoencephalitis.

	2013–2020 Cohort	2000–2010 Cohort	*p*-Value
Total No.	47	46	
Age (mean ± SD), years	62 ± 18	51 ± 19	<0.01
Female sex	14 (29.8)	14 (30.4)	>0.99
Time to diagnosis from presentation (mean ± SD), days	8.0 ± 8.6	7.1 ± 10.2	0.66
Inpatient mortality	12 (25.5)	18 (39.1)	0.16
CSF CrAg titer > 1024	16 (34)	10 (21.7)	0.17
Cryptococcemia	14 (29.8)	12 (26.1)	0.69
*C. gattii*	6 (12.8)	4 (8.7)	0.74
HIV infection	4 (8.5)	6 (13.0)	0.52
Hematologic disease/malignancy	7 (14.9)	4 (8.7)	0.36
Liver cirrhosis	4 (8.5)	5 (10.9)	0.74
Solid-organ transplantation	2 (4.3)	1 (2.2)	>0.99
Solid-organ malignancy	8 (17.0)	6 (13.0)	0.59
Autoimmune disease	5 (10.6)	1 (2.2)	0.20
Standard induction therapy	47 (100)	1 (2.2)	<0.01

Abbreviations: CrAg, cryptococcal antigen; CSF, cerebrospinal fluid; HIV, human immunodeficiency virus; SD, standard deviation.

## Data Availability

Data available on request.

## Supplementary Materials

The following supporting information can be downloaded at: https://www.mdpi.com/article/10.3390/jof8030287/s1, Figure S1: Patients with cryptococcal meningoencephalitis or cryptococcemia with identified sequence types and their residences; Figure S2: The geographical origins of *C. neoformans* VNI STs were obtained from the present study and the MLST database; Figure S3: Duration of follow up of the patients with cryptococcal meningoencephalitis.
