# Supplementary material for: Epidemiological and Clinical Characteristics, Antifungal Susceptibility, and MLST-Based Genetic Analysis of Cryptococcus Isolates in Southern Taiwan in 2013–2020
Source: J Fungi (Basel). 2022 Mar 11;8(3):287. doi: 10.3390/jof8030287 (PMC8951076; doi:10.3390/jof8030287)
Supplement: Supplementary file 1 [file jof-08-00287-s001.zip › jof-1633818-supplementary.pdf]

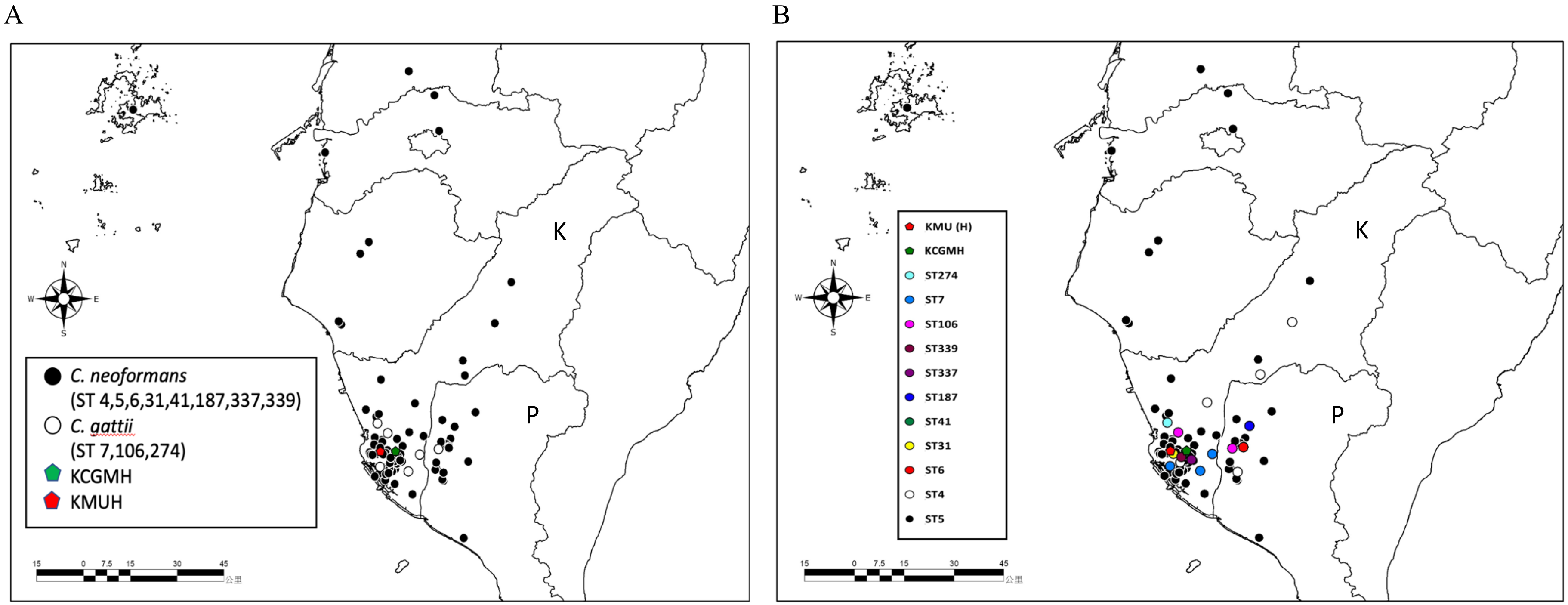

Supplementary Figure S1. (A) *Cryptococcus gattii* were identified from patients who lived in Kaohsiung and Pingtung. ST5 was identified in a patient from Penghu. (B) There were 8 *C. neoformans* and 3 *C. gattii* STs identified from patients who lived in Kaohsiung and Pingtung.

KCGMH, Kaohsiung Chang Gung Memorial Hospital; Kaohsiung Medical University Hospital (KMUH)

K, Kaohsiung; P, Pingtung

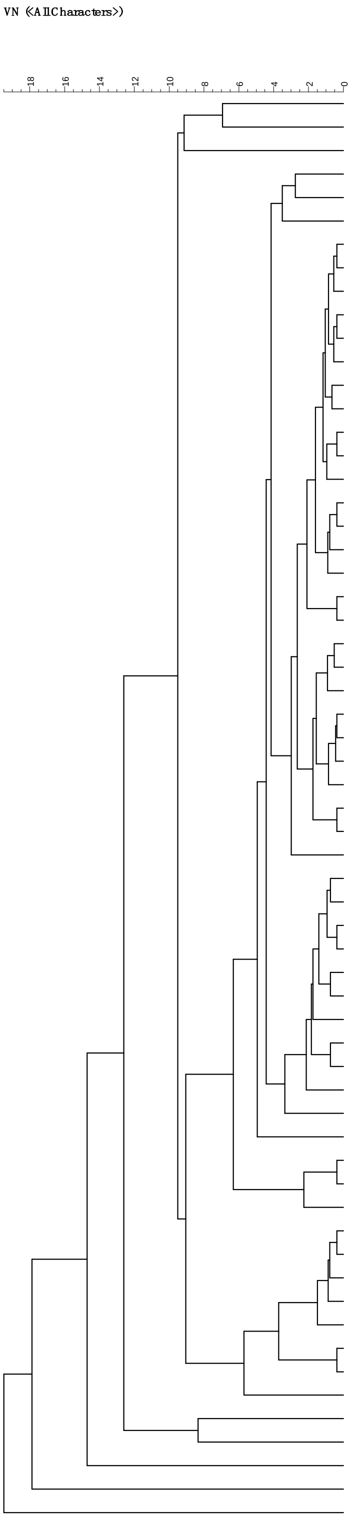

VN1

CAP59	GPD1	IGS1	LAC1	PLB1	SOD1	URA5	ST	Country
1	1	25	3	16	1	1	78	Uganda
1	1	8	3	23	1	1	238	South Africa
1	6	22	18	4	1	1	139	China
7	5	1	1	3	4	9	21	South Africa
1	7	3	1	4	1	9	36	South Africa
1	1	1	4	2	1	14	141	Thailand
1	3	1	5	2	1	1	5	Southern Taiwan,China,France,Germany,Italy,Japan,USA
1	3	1	4	2	1	1	337	Southern Taiwan,China
1	3	1	3	2	1	1	53	Thailand
1	1	1	5	2	1	1	81	Peru,Thailand
1	1	1	4	2	1	1	175	Thailand
1	1	1	3	2	1	1	174	India,Kuwait
1	1	1	3	4	1	1	3	Thailand
1	1	1	2	3	1	2	659	Congo Dem.Rep.
1	1	1	1	1	1	2	37	Peru
1	1	1	2	1	1	2	56	Italy
3	1	1	3	1	1	2	323	France
1	1	1	4	2	1	5	4	Southern Taiwan,Germany,India,Indonesia,Japan,South Korea
1	1	1	3	2	1	5	6	Southern Taiwan,Germany,India,Indonesia,Thailand,Uganda
1	3	1	4	2	1	5	137	Thailand
1	1	1	3	4	1	5	83	Vietnam
1	5	1	1	4	1	1	13	Botswana
1	6	1	1	4	1	1	22	South Africa
7	1	1	6	1	1	2	65	Italy
7	1	1	5	1	1	1	230	Japan
7	3	1	5	2	1	1	226	China
7	1	1	1	1	1	1	1	Peru,USA
7	1	1	1	1	1	2	2	France,Germany,Malaysia,Peru,South Africa,Tanzania,USA
7	1	1	2	1	1	2	23	France,Germany,Italy,Japan,South Korea,USA
7	3	1	2	1	1	1	138	Italy
7	5	1	3	3	1	1	69	Germany,Indonesia,Italy,Kuwait,Uganda
7	5	1	3	4	1	1	71	Germany,India,Italy
1	1	1	9	2	1	5	82	Thailand
1	1	10	3	2	1	1	31	Southern Taiwan,India,Japan,South Korea,Thailand
1	1	10	5	2	1	1	191	China
1	1	10	3	4	1	1	32	China,Japan,USA,Vietnam,South Africa
1	1	11	3	4	1	1	39	Uganda,USA
1	3	10	5	2	1	1	194	China
1	5	10	5	2	1	1	339	Southern Taiwan,Vietnam
1	1	10	4	2	1	5	190	Thailand
1	3	14	3	4	1	1	59	Italy
1	1	14	3	4	1	1	75	Italy
1	2	6	3	2	1	1	15	Brazil,USA
1	5	18	1	4	1	1	34	South Africa
1	3	1	5	2	12	2	202	South Africa
7	1	1	18	1	1	1	63	China,France,Germany,Italy,Japan,Uganda
7	1	1	18	1	1	2	67	Germany
1	1	1	18	1	1	2	58	Germany,Italy,South Africa,USA
1	23	10	3	4	1	1	93	India,Indonesia,Kuwait,Uganda
1	23	10	3	4	1	2	540	Brazil
1	23	10	3	2	1	1	187	Southern Taiwan,Uganda
1	23	10	5	4	1	1	195	China
1	23	10	3	4	1	5	177	Indonesia
1	26	1	5	2	1	1	186	China
1	25	1	5	2	1	1	359	China
1	23	10	18	4	1	1	319	China
7	1	1	18	1	24	1	64	USA
1	1	10	3	2	36	1	185	Thailand
17	21	28	13	14	1	16	127	South Korea
1	25	1	5	44	1	1	657	China
52	3	1	5	2	1	1	535	China

Supplementary Figure S2. The geographical origins of *C. neoformans* VNI STs were obtained from the present study and the MLST database.

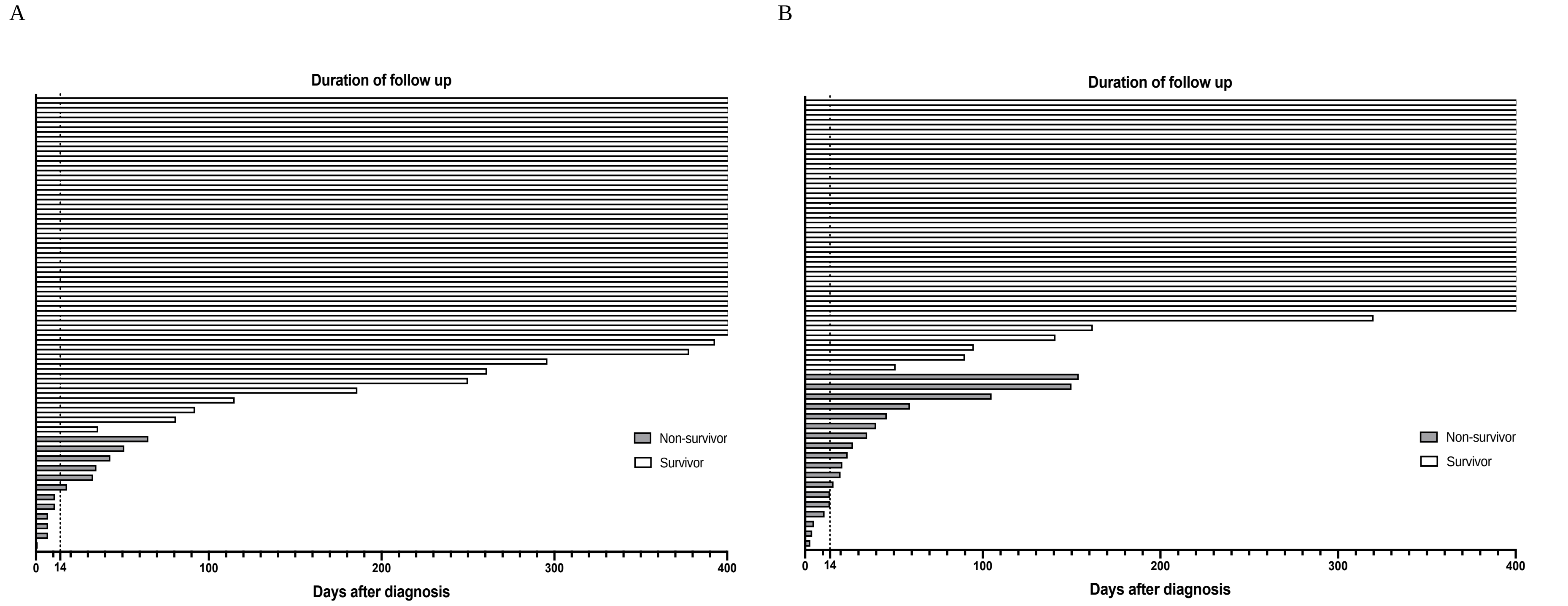

Supplementary Figure S3. Duration of follow up of the patients with cryptococcal meningoencephalitis during 2013-2020 (A) and 2000-2010 (B).
